# Supplementary material for: Ectopic Over-Expression of BjuAGL9-2 Promotes Flowering and Pale-Yellow Phenotype in Arabidopsis
Source: Plants (Basel). 2025 Nov 17;14(22):3502. doi: 10.3390/plants14223502 (PMC12656236; doi:10.3390/plants14223502)
Supplement: Supplementary file 1 [file plants-14-03502-s001.zip › plants-3922537-supplementary.pdf]

Supplementary Material

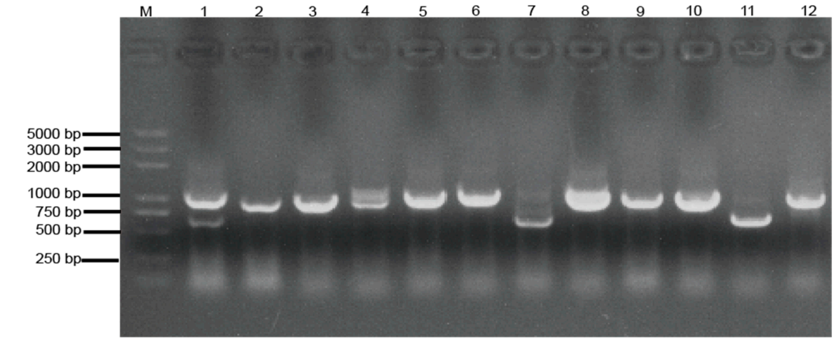

Figure S1. The PCR results of genes which may produce proteins interacted with BjuAGL9-2.

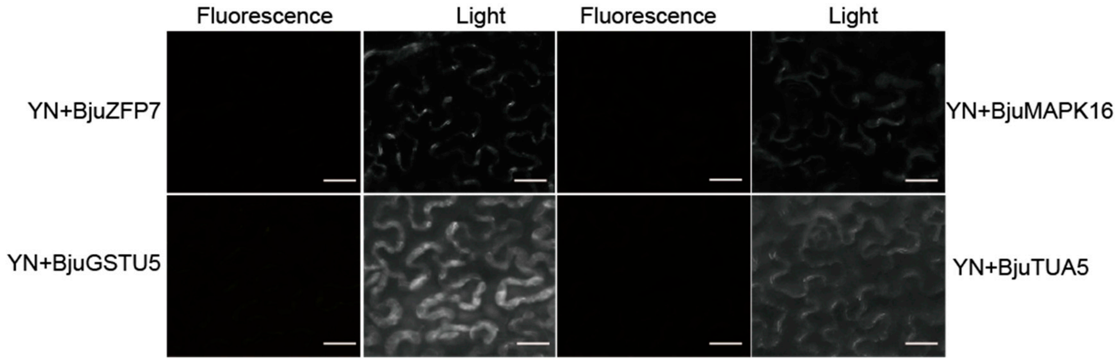

Figure S2. Bimolecular fluorescence complementation (BiFC) assays in tobacco leaf cells testing protein-protein interaction between BjuAGL9-2 with BjuTUA5, BjuZFP7, BjuGSTU5 and Bju-MAPK16. Fluorescence, yellow fluorescence signal; Light, bright light field; Bar=100 µm.

Table S1 Primer sequences used for Y2H, BiFC, qRT-PCR and sub-cellular location assays.

Primers names	Sequences	Primers names	Sequences
pGBKT7-BjuAGL9-2	5’-TCAGAGGAGGACCTGCATATGGGAAGAGGG AGAGTAGAATTG-’3	*dAtHEMA3*	5’-ATGGCGGTTTCTAATGCCTC-’3
	5’-TCGACGGATCCCCGGGAATTCTTACCCCATTT GAAGAATG-’3		5’-GTATCTGTCAATTGCAGGAG -’3
pGADT7-BjuANXAD1	5’-GTACCAGATTACGCTCATATGACATTGGCAAA GCAAGAGGC-’3		5’-ATGCCTGGAGGTGTGAATTC-’3
	5’-ATGCCCACCCGGGTGGAATTCTTAAGCATCA TCTTCACCG-’3	*dAtHEML1*	5’-ACAGCTGATATAACCATCTC-’3
pGADT7- BjuTUA5	5’-GTACCAGATTACGCTCATATGTATGGAAAGA AGTCTAAAC-’3	*dAtHEML2*	5’-ATGGCTGCGACGCTTACTGG-’3
	5’-ATGCCCACCCGGGTGGAATTCTCAGTAGTCT TCACCTTCGTC-’3		5’- CATCACCACTGGTTGTCCAC-’3
pGADT7- BjuZFP7	5’-GTACCAGATTACGCTCATATGACGGAATCTGA TGATGCCTC-’3	*dAtHEMB1*	5’- CTACACCCATCTTTAATGCTTC-’3
	5’-ATGCCCACCCGGGTGGAATTCTTAGAGTCTC AATGTAAGATC-’3		5’- CTTCAGGAACATTTCCAGC-’3
pGADT7- BjuGSTU5	5’-GTACCAGATTACGCTCATATGGCGGAGAAAG AAGAAGTGAAG-’3	*dAtHEMB2*	5’-ATGACGTCATCCATGTTTCG-’3
	5’-ATGCCCACCCGGGTGGAATTCCTAAGCGGAT CTGATTCTC-’3		5’- CTGAAGAGGCTGATCAATC-’3
pGADT7-BjuVA09G18110	5’-GTACCAGATTACGCTCATATGGCGAATCGATC CAGGG-’3	*dAtHEMC*	5’- ATGGATATTGCTTCGTCATC-’3
	5’-ATGCCCACCCGGGTGGAATTCTCAGCACTTG AACTTGAGGC-’3		5’- GAGGACTTCCCCTTGTGCC-’3
pGADT7- BjuWRKY11	5’-GTACCAGATTACGCTCATATGGCCGTCGATCT AATGCGC-’3	*dAtHEMD*	5’-ATGGCATTATTACTCCTTTC-’3
	5’-ATGCCCACCCGGGTGGAATTCTCAAGCCGAA TTAAACACAAA-’3		5’- GATGATTTGATTGTTCTTGC-’3
pGADT7- BjuRHY1A	5’-GTACCAGATTACGCTCATATGACGAGCGCATC AGAACTCTTC-’3	*dAtHEME1*	5’- ATGAGCTTATCATCGCCAAC-’3

	5’-ATGCCCACCCGGGTGGAATTCTCATTTAGCTATGGCTCTAC-’3		5’- GCTGGAGGACGACTTATGGC-’3
pGADT7-BjuVA01G03870	5’-GTACCAGATTACGCTCATATGGACTTTTTCCAGAAAGCC-’3	*dAtHEME2*	5’-ATGTCAATCCTTCAAGTCTC-’3
	5’-ATGCCCACCCGGGTGGAATTCTCACTTGATGAACCCTTCCG-’3		5’-CCTCCCAGCTTGCCTCATAAG -’3
pGADT7- BjuMAPK16	5’-GTACCAGATTACGCTCATATGCAGCCTGATCAACGCAAAAAG-’3	*dAtHEMF1*	5’-ATGGCTTCTCACTCGTCGAC-’3
	5’-ATGCCCACCCGGGTGGAATTCTTAGTACCAGCGGCTCATTG-’3		5’-TTGAGCAGCCCTAATCATAG -’3
NYFP-BjuAGL9-2	5’-GGGGACAAGTTTGTACAAAAAAGCAGGCTTCATGGGAAGAGGGAGAGTAGA-’3	*dAtHEMF2*	5’-ATGGCGTCTCACTCTTCGAC-’3
	5’-GGGGACCACTTTGTACAAGAAAGCTGGGTCTTACCCCATTTGAAGAATGG-’3		5’-GAAGAAGATTGAGTTTGATC-’3
CYFP-BjuTUA5	5’-GGGGACAAGTTTGTACAAAAAAGCAGGCTTCATGAGGGAGATCATAAGCAT-’3	*dAtHEMG1*	5’-ATGGAGTTATCTCTTCTCCG-’3
	5’-GGGGACCACTTTGTACAAGAAAGCTGGGTCTCAGTAGTCTTCACCTTCG-’3		5’-TTCGGAGCAGCATCAGGATG-’3
CYFP-BjuZFP7	5’-GGGGACAAGTTTGTACAAAAAAGCAGGCTTCATGACGGAATCTGATGATGC-’3	*dAtHEMG2*	5’-ATGCAAAATGGTTTGATTTG-’3
	5’-GGGGACCACTTTGTACAAGAAAGCTGGGTCTTAGAGTCTCAATGTAAGATC-’3		5’-CCATAAAAATGGTTCCAAC-’3
CYFP-BjuGSTU5	5’-GGGGACAAGTTTGTACAAAAAAGCAGGCTTCATGGCGGAGAAAGAAGAAG-’3	*dAtCHLH*	5’-ATGGCTTCGATTGTGTATTC-’3
	5’-GGGGACCACTTTGTACAAGAAAGCTGGGTCCTAAGCGGATCTGATTCTCTC-’3		5’-GGACGACGTAGACGATTTTC-’3
CYFP-BjuMAPK16	5’-GGGGACAAGTTTGTACAAAAAAGCAGGCTTCATGCAGCCTGATCAACGCAA-’3	*dAtCHLI1*	5’-ATGGCGTCTCTTCTTGGAAC-’3
	5’-GGGGACCACTTTGTACAAGAAAGCTGGGTCTTAGTACCAGCGGCTCATTG-’3		5’- TTTGAATCAAACTTCCCTAC-’3
p1300-BjuAGL9-2-GFP	5’-GAGAACACGGGGGACTCTAGAATGGGAAGAGGGAGAGTAG-’3	*dAtCHLI2*	5’-ATGGCGTCTCTTCTCGGAAG-’3
	5’-GCCCTTGCTCACCATTCTAGATTACCCCATTTGAAGAATGG-’3		5’-CGGATAAACAGGTCTTGCAC-’3
p1300- BjuTUA5-GFP	5’-GAGAACACGGGGGACTCTAGAATGAGGGAGATCATAAGCAT-’3	*dAtCHLD*	5’-ATGGCGATGACTCCGGTCGC-’3
	5’-GCCCTTGCTCACCATTCTAGATCAGTAGTCTTCACCTTCG-’3		5’- TTCCTGGCCAACAACTGCGG-’3
p1300- BjuZFP7-GFP	5’-GAGAACACGGGGGACTCTAGAATGACGGAATCTGATGATGC-’3	*dAtCHLM*	5’-ATGCCGTTTGCTCCTTCCTTG-’3
	5’-GCCCTTGCTCACCATTCTAGATTAGAGTCTCAATGTAAGATC-’3		5’- CCGCCAACTTCCTCCGCCTG-’3
p1300- BjuGSTU5-GFP	5’-GAGAACACGGGGGACTCTAGAATGGCGGAGAAAGAAGAAG-’3	*dAtCRD1*	5’-ATGGAACAGCTTTTCAACAC-’3
	5’-GCCCTTGCTCACCATTCTAGATAAGCGGATCTGATTCTCTC-’3		5’-CTTCGACCAAGCTCCTTGTAG-’3
p1300-BjuMAPK16-GFP	5’-GAGAACACGGGGGACTCTAGAATGCAGCCTGATCAACGCAA-’3	*dAtDVR*	5’-TTTAGTGGTTGGTTCAACTG-’3
	5’-GCCCTTGCTCACCATTCTAGATTAGTACCAGCGGCTCATTG-’3		5’- GACACAACAACATCAATACC-’3
p1300-GFP	5’-GAGAACACGGGGGACTCTAGA-’3	*dAtPORA*	5’-CATTCAAAGAGTCTAGTCTG-’3
	5’-GCCCTTGCTCACCATTCTAGA-’3		5’-CTAGCCCTGAAGAAGCTCCCG-’3
dBjuAGL9-2	5’-ATGGGAAGAGGGAGAGTAG-’3	*dAtPORB*	5’-ATGGCCCTTCAAGCTGCTTC-’3
	5’-TAACGCTCCTTAAGCTTAAG-’3		5’-CGTTTTCTTGCCGTCCAC-’3
dBjuACTIN2	5’-TCCAAGCTGTTCTCTCCTTGTAC-’3	*dAtPORC*	5’-ATGGCTCTCCAAGCTGCCTATTC-’3
	5’-GTTGTGGTGAACATGTAACCTCTC-’3		5’- TGCAGTGCCTTTTCTCTCAG-’3
dBjuTUA5	5’-ATGAGGGAGATCATAAGC-’3	*dAtCHLG*	5’-ATGACTTCGATTCTCAACAC-’3
	5’-GCTGACGGTACGTTCCGGT-’3		5’- GGTTAATGCTTGAACCAC-’3
dBjuZFP7	5’-ATGACGGAATCTGATGATGC-’3	*dAtCAO*	5’-ATGAACGCCGCCGTGTTTAG-’3
	5’-GCGTTCTCGCTTGTGAGCG-’3		5’- CTTTCCTTTATAAGGAGGAG-’3
dBjuGSTU5	5’-ATGGCGGAGAAAGAAGAAG-’3	*dAtFT*	5’-GTCTATAAATATAAGAGACC-’3
	5’-GGATTCTGTTTCCAAGTCTC-’3		5’-CGGAGGTGAGGGTTGCTAGG-’3
dBjuMAPK16	5’-ATGCAGCCTGATCAACGCA-’3	*dAtSOC1*	5’-CTCTTGGGAGAAGGCATAGG-’3
	5’-CTTGATCTCAACGATGTC-’3		5’-CCTCTTCCAGTACTTTCTTG-’3
dATTUA5	5’-ATGAGGGAAATTATTAGCAT-’3	*dAtLFY*	5’-GGATCCTGAAGGTTTCACG-’3
	5’-GTTGACGGTAAGTACCAGTAC-’3		5’-CCTTCATACCCACAAGCGTG-’3
dAtZFP7	5’-ATGACGGAATCTGATGATGC-’3	*dAtCO*	5’-GTTGAAACAAGAGAGTAACG-’3
	5’-CCAAGAGCTTGAGAGCTATA-’3		5’-GCATAGAGAGGCATCATCTG-’3
dAtGSTU5	5’-ATGGCTGAGAAAGAAGAAGTG-’3	*dAtFLC*	5’-CTAGAAATCAAGCGAATTG-’3
	5’-GTTCATCGACGAGTTTAGC-’3		5’- CCATAGTTCAGAGCTTTTG-’3

dAtMAPK16	5'-GATCACCGCAAAAAGTCATC-'3<br>5'-CGGCAACAAGATGTGTTTG-'3	*dAtFUL*	5'- GGGAAGAGGTAGGGTTCAGC-'3<br>5'-CGTCTCGGCCAACAAGTTG-'3
dAtACTIN	5'-TCCAAGCTGTTCTCTCCTTGTAC-'3<br>5'-GTTGTGGTGAACATGTAACCTCTC-'3	*dAtSVP*	5'-GGCGAGAGAAAAGATTCAG-'3<br>5'-GCTGATCAAGCTTCTCCAAG-'3
dAtHEMA1	5'-ATGGCGGTTTCAAGTGCTTTC-'3<br>5'- GCTGCAGAGTTCTTGAGTTG-'3	*dAtAP1*	5'-GGGGTAGGGTTCAATTGAAG-'3<br>5'-CTCAGGTGCAATAAGCTGTC-'3
dAtHEMA2	5'- ATGGCGGTTTCTAGCGCCTTC-'3<br>5'-TTAGTGTATCGGTCAATAGC -'3		
